# Intrathoracic intestinal diverticulum in a late presenting congenital bilateral diaphragmatic hernia: a case report

**DOI:** 10.1186/1752-1947-7-290

**Published:** 2013-12-30

**Authors:** Ruth Gómez-Rosales, Santiago Petersen-Morfín, Miguel Haro-García, Alejandra Ortiz-González, Alejandro Porras-Ruiz, Roberto González-Chávez

**Affiliations:** 1 Department of Surgery, Hospital Civil de Guadalajara Fray Antonio Alcalde, Calle Hospital 278, Guadalajara, CP 44280, Mexico

**Keywords:** Bilateral congenital diaphragmatic hernia, Congenital diaphragmatic hernia, Late presenting diaphragmatic hernia, Morgagni-Larrey hernia

## Abstract

**Introduction:**

Hernias comprise 3% of all defects of the diaphragm. Bilateral hernias are extremely rare and usually occur in children. Here we present a case report of a bilateral Morgagni-Larrey diaphragmatic hernia with an intrathoracic intestinal diverticulum and late presentation. To the best of our knowledge this is the first report of this type.

**Case presentation:**

A 37-year-old Hispanic man was admitted to our emergency department with a 4-day history of obstipation, abdominal pain, distension, nausea, and vomiting. During the initial evaluation, chest and abdominal X-rays were performed, which revealed intestinal displacement into his right and left hemithorax. During laparotomy, a Morgagni-Larrey hernia with a sac was found. His small bowel with a large diverticulum, transverse colon, descending colon, and epiploic fat were herniated into his thorax. Tissues were returned to his abdominal cavity and the hernia defects were corrected with running non-absorbable sutures. He had no postoperative complications.

**Conclusions:**

Bilateral congenital diaphragmatic hernias remain extremely rare. However, they should be considered in adult patients with intestinal obstruction even when respiratory symptoms are absent. This is the first description of a patient with a prolapsed intestinal diverticulum and bilateral diaphragmatic hernias.

## Introduction

Four types of diaphragmatic defects are documented. Bochdalek’s hernias represent 90% of cases, and Morgagni’s hernias comprise 2% to 3% [1]. In most cases, diaphragmatic hernias occur on the right side (10:1 ratio, right: left) [2]. When the defect is bilateral it is known as a Morgagni-Larrey type, which represents 0.12% of congenital diaphragmatic hernias [3]. This type of hernia is commonly diagnosed in pediatric patients, and late presentation is extremely rare [4]. Importantly, an intestinal diverticulum and bilateral herniation have never been reported together.

**Figure 1:**
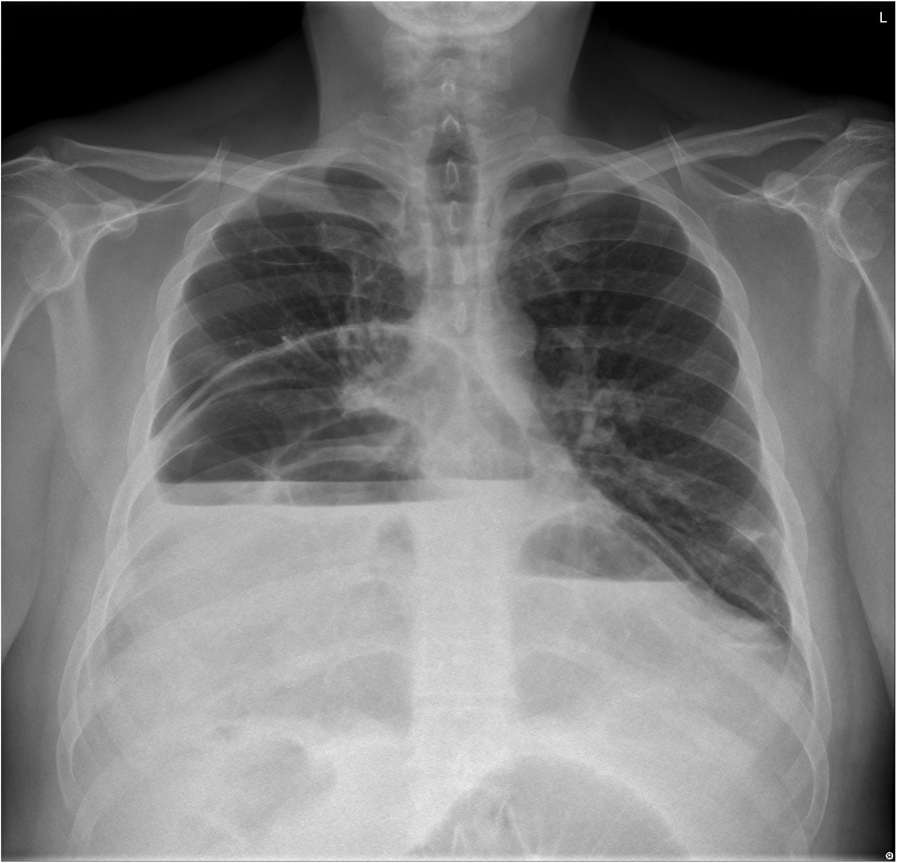
**Preoperative X-ray.** X-ray of the chest revealed air-fluid levels on right and left hemithorax.

**Figure 2:**
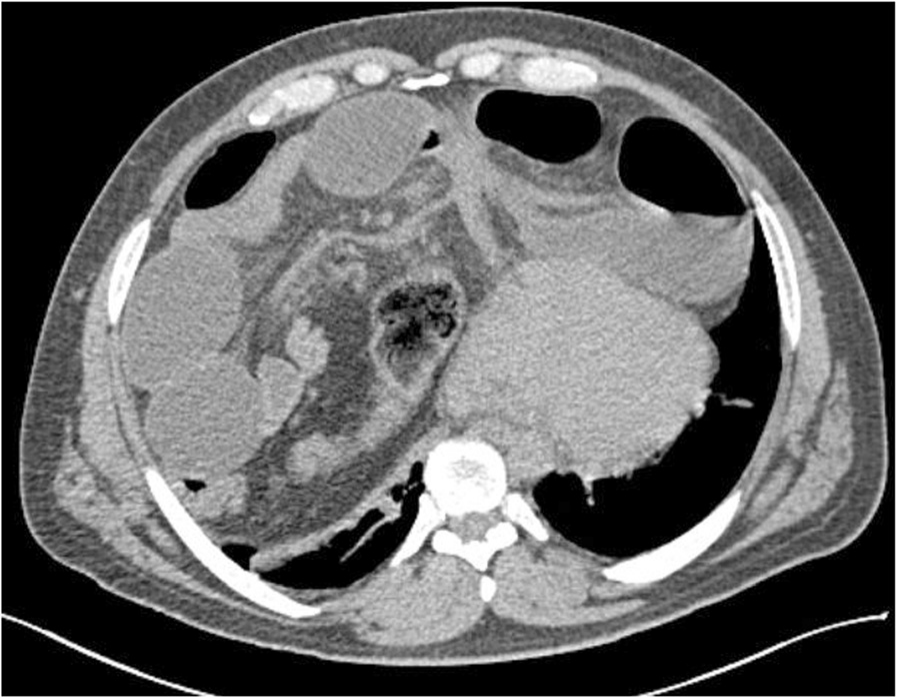
**Preoperative computed tomography.** Preoperative computed tomography scans showed herniation of the intestinal content into the thorax. Cardiac deviation was also shown.

**Figure 3:**
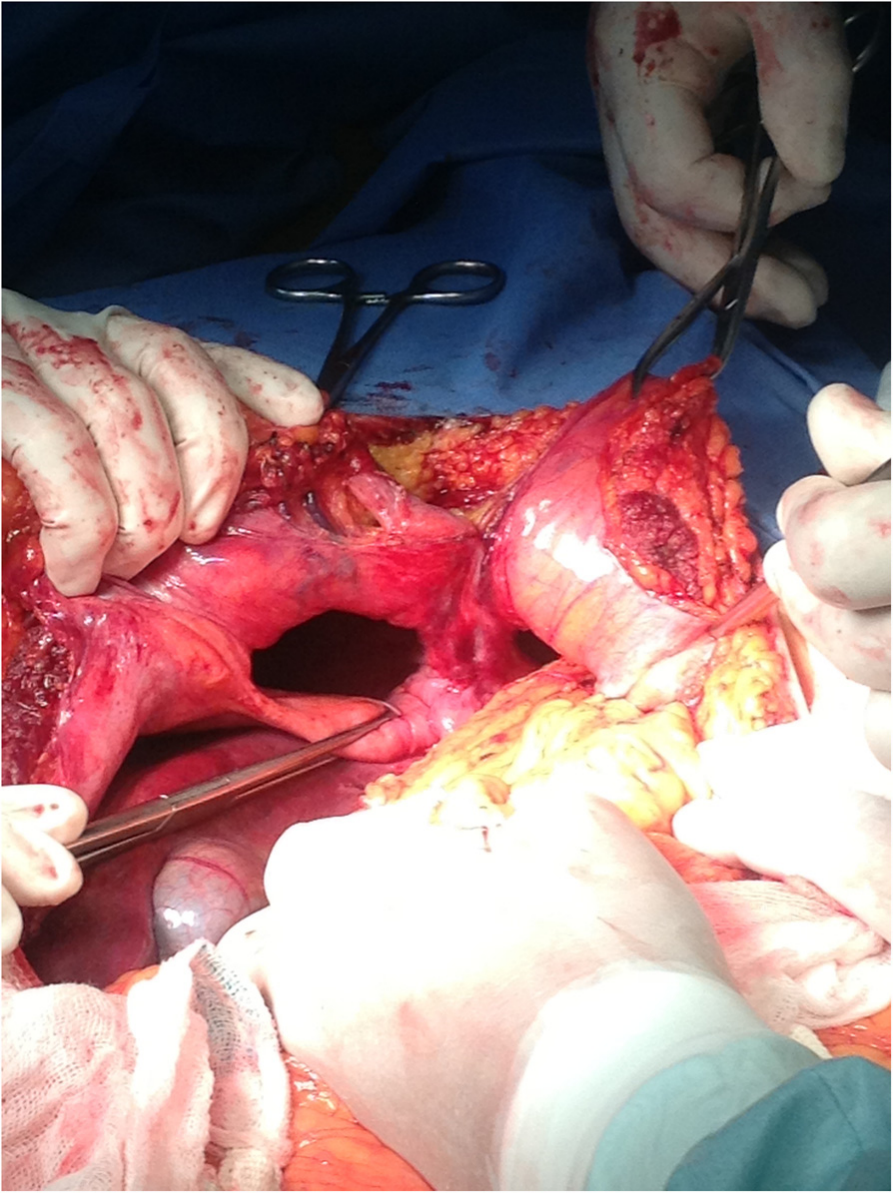
**Operative findings.** Bilateral diaphragmatic defects were shown after reducing the intestinal content to the abdominal cavity.

**Figure 4:**
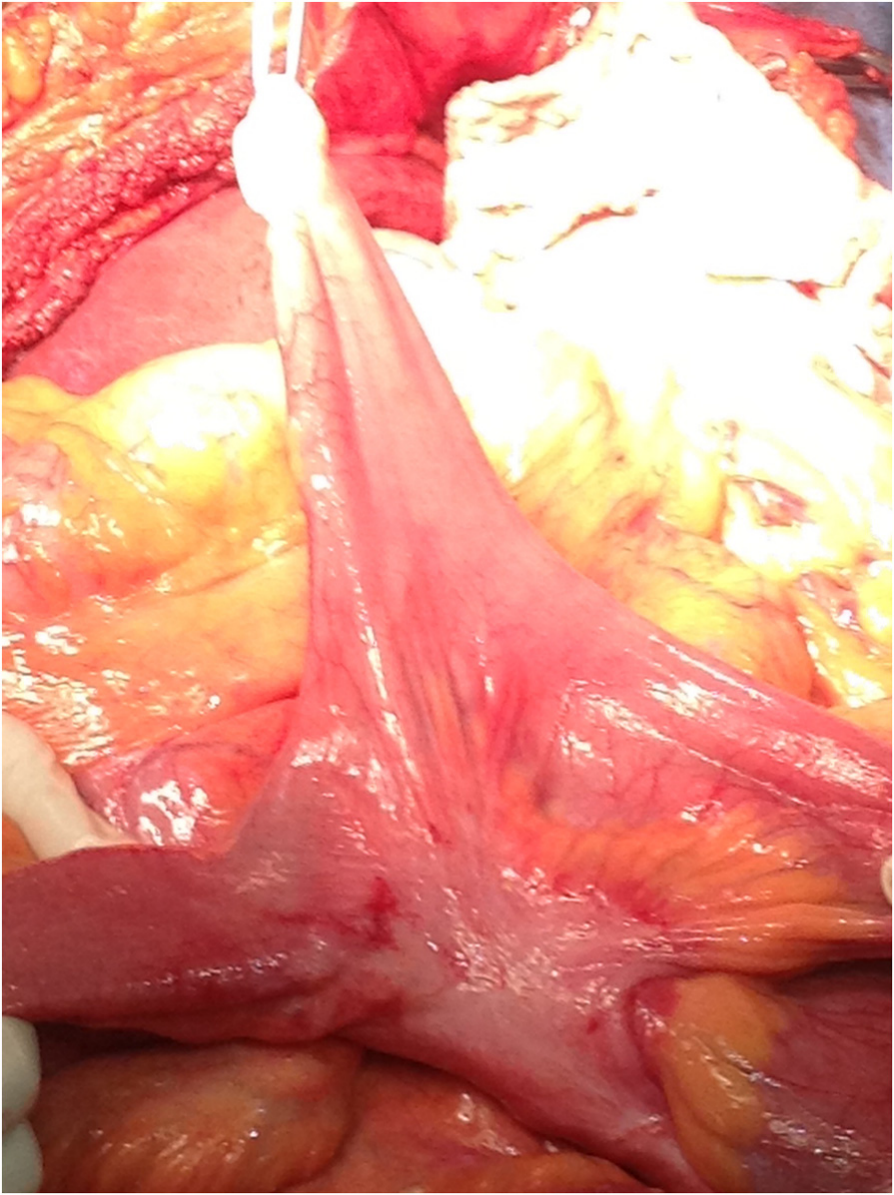
The intestinal diverticulum was shown after being reduced back to the abdominal cavity.

**Figure 5:**
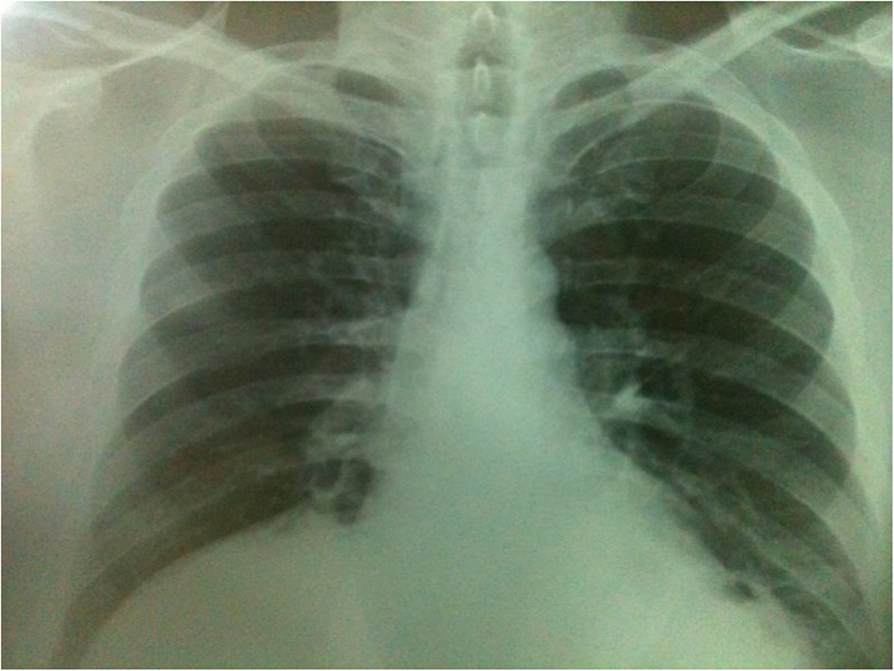
Postoperative X-ray taken the day after the procedure.

## Case presentation

A 37-year-old Hispanic man who has human immunodeficiency virus was admitted to our emergency department with a 4-day history of obstipation, abdominal pain, distension, nausea, and vomiting. He did not report any episodes of shortness of breath, however, he reported transient tachycardia when lying on his right or left side. On physical examination, abdominal distension in his right upper quadrant was observed. Bowel peristalsis was noted during right chest auscultation. He complained of epigastric pain on palpation.

During the initial examination, chest and abdominal X-rays revealed intestinal displacement into his right and left hemithorax and air-fluid areas in both his abdomen and thorax (Figure 1). Computed tomography (CT) images revealed a large segment of small bowel herniating into his left hemithorax (Figure 2) and a portion of transverse colon herniating into his right hemithorax. Right-side cardiac deviation was also observed. Laboratory tests returned results within normal ranges.

He was diagnosed with intestinal obstruction caused by herniation through the diaphragm, and surgery was performed. During laparotomy, a bilateral Morgagni-Larrey hernia with a sac was found (Figure 3). The right side of his diaphragm showed a defect with a 7cm diameter, and 130cm of small bowel was herniated into his thorax. When his small bowel was manually returned to the abdominal cavity, a large diverticulum was noted 160cm from the ileocecal valve (Figure 4). His transverse colon, his descending colon, and epiploic fat herniated through a 4cm defect on the left side of his diaphragm. After returning the herniated content to his abdominal cavity, the hernia defects were closed with running non-absorbable sutures. He had no postoperative complications (Figure 5).

## Discussion

A Morgagni hernia was first reported by Morgagni in 1769 [5]. Morgagni-Larrey hernias, congenital hernias, are extremely rare. They are characterized by bilateral herniation of the abdominal organs into the thorax, and they usually present with respiratory distress in neonates in the first few hours of life. They can also be detected in the prenatal period [6]. Postnatal survival rates are 70% to 98%. The survival rate in patients who present late is approximately 100% [7,8]. In adult patients, symptoms are predominantly gastrointestinal and respiratory, however, 35% are asymptomatic [9]. In our patient, the main symptom was intestinal obstruction, and he lacked respiratory symptoms despite the large segment of herniated bowel. The transient tachycardia in this patient may have been caused by cardiac compression. Congenital hernias are typically identified with X-rays, which can reveal air and fluid levels, the cardiophrenic angle, simulating pleural effusions, or paracardiac masses [10,11]. CT and magnetic resonance imaging are used to confirm diagnoses when there is liver herniation and the possibility of mediastinal tumors [12].

For treatment, surgery should always be considered due to the risk of strangulation [13]. The abdominal approach is sometimes preferable, especially when other intra-abdominal procedures may be needed [14]. Laparoscopic approaches are preferred for elective procedures [15]. In this particular patient with acute intestinal obstruction, laparotomy was preferred. During the procedure, an intestinal diverticulum was discovered and reduced. To the best of our knowledge, there are no previous case reports of Morgagni hernia with a concomitant diverticulum, although they share congenital origins. Finally, defects should be closed by fixing a mesh with a hernia stapler or by direct approximation. Prognosis after surgery using these methods is generally good.

## Conclusions

Although late presentation of congenital bilateral diaphragmatic hernias is rare, they do occur in adult patients. As reported here, patients can present with intestinal obstruction and lack respiratory symptoms. This is the first report of a Morgagni-Larrey hernia occurring with a prolapsed intestinal diverticulum. Laparotomy and hernia closure with running non-absorbable sutures produced positive results with no postoperative complications.

## Consent

Written informed consent was obtained from the patient for publication of this case report and accompanying images. A copy of the written consent is available for review by the Editor-in-Chief of this journal.

## Competing interests

The authors declare that they have no competing interests.

## Authors’ contributions

RGR and SPM were involved in the literature review and were major contributors to manuscript preparation. MHG and RGC treated the patient and were responsible for manuscript review. AOG and APR helped with data collection and to review the manuscript. All authors read and approved the final manuscript.
